# Effects of Fasting on the Physiological and Psychological Responses in Middle-Aged Men

**DOI:** 10.3390/nu15153444

**Published:** 2023-08-03

**Authors:** Krzysztof Stec, Karol Pilis, Wiesław Pilis, Paweł Dolibog, Sławomir Letkiewicz, Alicja Głębocka

**Affiliations:** 1 Collegium Medicum, Jan Dlugosz University in Czestochowa, ul. Waszyngtona 4/8, 42-200 Częstochowa, Poland; k.stec@ujd.edu.pl (K.S.); w.pilis@ujd.edu.pl (W.P.); s.letkiewicz@ujd.edu.pl (S.L.); 2 Department of Biophysics, Faculty of Medical Sciences in Zabrze, Medical University of Silesia, 40-752 Katowice, Poland; pawel.dolibog@sum.edu.pl; 3 Institute of Health Sciences, University of Opole, 45-401 Opole, Poland; 4 Department of Economy in Opole, WSB Merito University in Wroclaw, 53-609 Wroclaw, Poland; alicja.glebocka@wsb.opole.pl

**Keywords:** fasting, men, psychological variables, physiological indicators

## Abstract

Tracking changes in the body during fasting takes into account indicators of mental well-being and physiological parameters. The aim of the study was to measure psychological and physiological reactions, along with their mutual relations, caused by 8 days of water-only fasting. Fourteen men aged 35 to 60 participated in the study, divided into two groups, younger and elder. In addition to physiological parameters, psychological data were collected using four different tests. The obtained results confirmed reduction in body weight, systolic blood pressure, resting diastolic blood pressure and glucose level, and increase in resting heart rate, cortisol and β-hydroxybutyrate concentration. However, no significant psychological changes were observed under the influence of fasting intervention. A significant interaction effect occurred for the state anxiety variable determined before and after the fasting intervention for both groups. Moreover, negative correlations between physiological (cortisol) and psychological factors of subjectively assessed stress were revealed. The only effect on cognitive ability was seen when responding to simple tasks. The study confirmed the beneficial effect of 8 days of water-only fasting on physiological variables without affecting mental well-being. The relatively high level of well-being after fasting intervention was independent of the physiological indicators of stress.

## 1. Introduction

Fasting is often practiced in the modern world. A relatively mild form of fasting includes caloric restriction, which has beneficial health effects in patients with non-communicable and chronic diseases [1], as well as in people who want to reduce excessive body mass [2]. The most restrictive form of fasting involves complete cessation of all food intake for several days to several weeks, apart from consuming any amount of water [3]. This restrictive form of fasting in the first phase can also bring beneficial health effects until the threshold of hormesis is reached, i.e., the balance between long-term benefits and emerging damage caused by insufficient caloric intake [4]. Determining this moment during fasting is difficult and ambiguously described in the literature, as it is characterized by high individual variability. However, already in the initial stages of fasting, before reaching the threshold of hormesis, mild ketosis develops, which provides ketone bodies as alternative sources of energy for the body while decreasing glucose resources that also protect the metabolism of the central nervous system [5,6,7,8].

It turns out that one of the ketone bodies, i.e., β-hydroxybutyrate (β-HB), also plays an important role in cell signaling, by means of which adaptive neuronal responses to various stressors, such as fasting or physical exercise, can be generated [9,10,11,12].

It has also been described that significant hormonal and metabolic changes of varying severity have been observed during fasting [13]. One such change is the stimulation of the hypothalamic–pituitary–adrenal (HPA) axis, which is co-responsible for inducing the stress response [14,15,16,17,18]. The stress response is a complex combination of three interplays, i.e., the sympathetic nervous system, the HPA axis and the subjective emotional experience of the individual [19]. The degree of HPA activity is determined by measuring the concentration of one of the glucocorticoids, i.e., cortisol in the serum, the concentration of which significantly increases during fasting [20,21,22,23,24]. Increased concentrations of glucocorticoids also affect a number of processes, including memory and emotional arousal and processing [25,26]. Questions about the scope and dynamics of these changes, as well as about the most effective type of fasting, still do not have clear answers. The most common measurements are the psychological effects of fasting in specific rhythms interrupted by periods of normal food consumption, e.g., every other day or two days of fasting per week. Some of them prove that short-term fasting may increase negative emotions, such as the occurrence of depression, anxiety, tension, anger, irritability and fatigue, while reducing the strength of positive emotions. These unfavorable affective changes coexist with lower work efficiency [27]. Emotional changes in the form of increased anger and aggression were also observed during a 48 h fast in a group of amateur weightlifters, with no changes in mental flexibility and set shifting [28]. In addition, in a group of older, obese women, reduced mental flexibility was observed after such a fasting session [29]. In turn, other studies have shown positive changes in mood and perceived work performance among short-term fasting people, which, according to the authors, did not result directly from the fasting itself but from the distraction that accompanies fasting [30]. The effect of an 18 h fast among healthy women turned out to be increased irritability on the one hand and positive changes in self-esteem, such as a sense of achievement, pride and control, on the other hand [31]. In the literature on the subject, there are also reports that there is no significant difference in terms of experienced affective states between fasting and non-fasting days and between fasting and non-fasting people [28,32].

Research on the psychological consequences of long-term fasting has also provided interesting data. It turns out that during such fasting, changes in affective states (anxiety, depression, fatigue) took the shape of “U” or “∩” [33]. The inflection point of the described curves coincided with the moment of change in carbohydrate-fueled metabolism to the use of ketone bodies by the human organism. In addition, a significant effect of the duration of fasting on the emerging depression and feeling depressed was found, which reached the highest level on the third day after the end of the fasting, and experiencing anxiety, the highest intensity of which was recorded on the sixth day after the end of the fasting [33].

Important data on the psychological aspects of a 7-day fast were provided by pilot studies, which showed an increase in motivation and an improvement in mood among the respondents but at the same time an increase in irritability and internal tension. Interestingly, although the subjects experienced symptoms such as headaches, nausea, unpleasant body odor, poor concentration and intense dreams, these people unanimously claimed that they felt mentally strengthened after a week of fasting [34].

The mechanism of the impact of fasting on mental conditions is still far from being explained; therefore, in the presented study, an attempt was made to determine to what extent an 8-day water-only fast affects levels of stress and anxiety, and the efficiency of cognitive processes, taking into account physiological conditions.

Hypothesis. Due to the complexity of the issues posed for the purpose of the paper, two hypotheses were put forward.

**Hypothesis** **1.** *Physiological parameters undergo significant changes under the influence of 8 days of water-only fasting and physical effort.*

**Hypothesis** **2.** *The applied 8 days of water-only fasting have no significant effect on the variables describing the mental state of the examined men.*

## 2. Materials and Methods

### 2.1. Participants

Fourteen male volunteers (ages 35–60) were enrolled in the study. The surveyed men were recruited randomly by placing an advertisement in the mass media. Only those subjects who had never been diagnosed with mental disorders, especially eating disorders, and had never been treated psychiatrically could be included in the group. An important condition for qualifying for the study group was participation in fasting at least 4 times, lasting not less than 3 days in each case. Among the examined group of men, there were even those who had experienced a single complete fasting lasting 42 days, consuming only mineral water ad libitum. Such a significant fasting experience allowed the test men to tolerate the 8 days of water-only fasting without much difficulty. At that time, the volunteers were in excellent psychophysical condition during the day, they fell asleep quickly and, although they slept less, they felt comfortable, and their thoughts after the first day of fasting did not turn to the desired food consumption. The subjects thought positively; they were strongly motivated to participate in the next fasting session in their lives because they expected that the 8 days of water-only fasting would improve their health, which they had already experienced during previous fasting sessions. During the 8 days of water-only fasting, each of the men stayed at their home, but they kept in daily telephone contact with the doctor supervising the study, the psychologist participating in the study and the research organizational manager. In addition, for 8 days before the tests, these people maintained telephone contact with the men who were later examined. The volunteers participating in the research were government officials, business consultants and academics. During the 8 days of water-only fasting, the volunteers fulfilled their normal occupational duties, and, therefore, the effect of fasting deprivation could be demonstrated by comparing the results obtained before and after the fast. Volunteers were familiar with their eating routine after completing the 8 days of water-only fasting but were reminded in detail of their eating habits during this period. The observation of the study participants conducted for a period of 2 weeks after the end of the fasting intervention showed their excellent mental and physical conditions. The subjects did not show any symptoms of cognitive, emotional or behavioral disorders. They did not report any ailments related to psychophysical functioning and physiological changes. The inclusion criteria for the study were: (1) previous fasting practice and no fasting within the last 6 months; (2) non-smoker, non-drinker of alcohol or user of drugs; (3) no weight-loss diet in the last 6 months; (4) no evidence of any acute or chronic physical or mental illness; (5) no eating disorder and no previous head injury.

Since the examined men differed significantly in age, they were divided into 2 groups: younger (n = 6, age 38.5 ± 9.09 years) and elder (n = 8, age 54.00 ± 5.55 years). The division into 2 age groups was important due to the fact that, with age, there is a slower rate of metabolism, which results in a decrease in the efficiency of the body’s physiological functions. These changes are particularly intensified under caloric restriction as then there is a large reduction in energy expenditure which is greater than the loss of lean and fat body mass can explain [35]. The age of 48 was arbitrarily set as the limit of such a division.

The surveyed men were informed about the possible negative effects of the applied fasting intervention, which could be life threatening, and then gave their written consent to participate in the study.

The Ethical Committee for Scientific Research of the Jan Długosz University in Częstochowa (Poland; KE-0/1/2019; 5 March 2019) approved the research protocol in accordance with the requirements of the Helsinki Declaration.

### 2.2. Procedure

Male volunteers reported to the laboratory in the morning in the fasting state. At the beginning, the examined men, under the supervision of a psychologist, completed the following tests: Perceived Stress Scale (PSS), State-Trait Anxiety Inventory (STAI), Color Trail Test (CTT-1, CTT-2) and Cattell’s Fluid Test, Scale 2 (CFT-20R). After 10 min of rest, blood pressure and heart rate were measured. These variables of the circulatory system were recorded because they reflect the efficiency of this system, which is important both for somatic and mental human behavior. Then, blood was taken from the cubital vein to determine the concentrations of glucose, cortisol and β-hydroxybutyrate. These metabolites are especially important when observing the effects of fasting or starvation. Optimal blood glucose concentration corresponds to proper brain nutrition, and such a situation occurs in the conditions of using a mixed diet, which was the case in the volunteers we studied before fasting [36]. In fasting conditions, however, blood glucose concentration decreases, and brain nutrition is increasingly dependent on ketone bodies, of which β-hydroxybutyrate is an important representative. Therefore, it is interesting whether the change in the manner of brain nutrition affects the functioning of the psyche of people subjected to such influence, mainly in terms of subjectively perceived stress and increased fear and anxiety, as well as in terms of cognitive functions and psychomotor performance. We defined this metabolite in our study also because it is an important indicator of the body’s energy security during fasting, and a significant increase in its concentration in serum is an unquestionable proof of the reliability of maintaining the state of fasting by the subjects [37,38]. It should also be mentioned that the concentration of cortisol in the serum was determined because it is a commonly accepted exponent of the stress reaction [39]. As the presented research is of a pilot study nature, we considered the physiological variables listed above to be sufficient. They are of key importance for characterizing the stressful situation, which was the 8 days of water-only fasting. After that, the subjects underwent somatic measurements, and their age was recorded. In the next stage, the men were subjected to an exercise test of gradually increasing intensity on an ergometer. The test was started with a load of 30 W, which was increased every 3 min by 30 W until the individual maximum load was reached. In the last minute of the maximum load, blood pressure and heart rate were measured, and blood was collected to determine the concentrations of glucose, cortisol and β-hydroxybutyrate. After completing these activities, the men under study began 8 days of water-only fasting, during which they consumed only arbitrary amounts of the same mineral water. During the fasting, the men were under constant medical care. During the fasting, the surveyed men performed their daily work just as before the fasting intervention. For safety, the study participants remained in constant telephone contact with the medical doctor so that medical assistance could be provided in the event of any critical situation. After 8 days of fasting, the research procedures were repeated according to the scheme described above.

### 2.3. Measurements

#### 2.3.1. Anthropometric Measurements

The participants’ age, body height and other somatic variables, i.e., body mass, body fat, fat-free mass, total body water and body mass index, were determined using the Tanita TBF 300A body composition analyzer (Tanita, Amsterdam, The Netherlands) (Table 1).

#### 2.3.2. Analysis of Blood Samples

Blood samples from the cubital vein were drawn for determination of glucose, cortisol and β-hydroxybutyrate concentrations. Biochemical analysis was achieved with the blood of the fasting men, which was collected in EDTA-treated tubes. After separating the serum, glucose concentration was determined using diagnostic kits (Glucose GOD-POD, Liquid, Spinreact, Spain), reference values, serum or plasma: 60–110 mg/dL; ~3.33–6.10 mmol/L. The serum cortisol concentration was measured using tests from MAGLUMI (Cortisol-CLIA, Shenzhen New Industries Biomedical Engineering Co., Ltd., Shenzhen, China), reference values in serum: 52–350 ng/mL. Moreover, β-hydroxybutyrate concentration was determined using the RANBUD diagnostic kit from Randox, Laboratories Ltd., Crumling, UK, reference values in serum: 0.03–0.3 mmol/L.

#### 2.3.3. Assessment of Cardiovascular Variables

Systolic blood pressure and diastolic blood pressure were measured with the use of a manual sphygmomanometer. Heart rate was measured using an electronic recorder (Ergo Card-Belgium). The exercise test was performed on a Lode Excalibur cycle ergometer.

#### 2.3.4. Assessment of Psychological Variables

The level of stress was measured using the Perceived Stress Scale (PSS) [40], a questionnaire in Polish adapted by Jurczyński and Ogińska-Bulak [41]. The scale consists of 10 statements related to subjectively assessed symptoms of stress.

Measurement of State-Trait Anxiety Inventory (STAI) [42] was achieved using a questionnaire in Polish adapted by Sosnowski et al. [43]. The STAI-Y1 scale is used to measure state anxiety, i.e., currently experienced anxiety, while STAI-Y2 measures anxiety as a trait and refers to generally experienced anxiety.

Measurement of Color Trail Test (CTT-1, CTT-2) [44,45] was achieved using a Polish adaptation by Łojek and Stańczak [46]. The test is used to measure executive functions (attention, information processing and monitoring one’s own behavior) and detection of psychological disorders.

Measurement of Cattell’s Fluid Test, scale 2 (CFT-20R) [47], was achieved using a Polish adaptation by Stańczak [48]. This test is designed to determine fluid intelligence and consists of discovering logical relationships between different figures. Tasks are non-verbal, and their execution is time limited.

### 2.4. Statistical Analyses

The data obtained in the study are presented as arithmetic means (M) and standard deviations (±SD). Analyses of the impact of fasting on selected physiological parameters (blood pressure, heart rate, concentration of glucose, cortisol, β-hydroxybutyrate), the level of perceived stress (PSS 10) and anxiety (STAI), as well as the efficiency of cognitive processes (CTT-1 and CTT-2 and CFT-20R), were performed using repeated-measures analysis of variance and the age grouping factor (younger vs. elder subjects). Moreover, post hoc analysis with the Scheffe test were carried out. Somatic data analyses were performed using the *t*-test for paired data.

In addition, Pearson’s correlation analyses were carried out for indicators assessing objectively (C) and subjectively the level of stress (PSS), anxiety (STAI) and cognitive performance (CTT-1 and CTT-2 and CFT-20R). Values at *p* < 0.05 were considered statistically significant. Calculations were made using Statistica, version 13.

## 3. Results

### 3.1. Physiological Results

The results regarding changes in physiological indicators caused by 8 days of water-only fasting showed statistically significant changes for almost all parameters measured at rest and after exercise included in the study. Resting blood pressure and post-exercise systolic blood pressure decreased, but heart rate measured at rest increased (Table 2).

Body mass also decreased, which before fasting was 79.75 ± 10.85 kg and after was 74.19 ± 10.79 kg (relative percentage change body mass equaled 8.97%), F(2.12) = 240.35, *p* < 0.001, η^2^ = 0.95. Measurements of blood G levels also showed a statistically significant reduction after fasting (Table 3).

Measurements of the cortisol level before (308.58 ± 87.36 ng/mL) and after fasting (487.30 ± 140.07 ng/mL) measured at rest showed statistically significant differences, F(1.12) = 22.193, *p* < 0.001, η^2^ = 0.649. At the same time, there were not statistically significant differences for the age groups: younger (414.27 ± 99.21 ng/mL) and elder (385.69 ± 126.70 ng/mL), F(1.12) = 0.306, *p* > 0.05.

**Figure 1:**
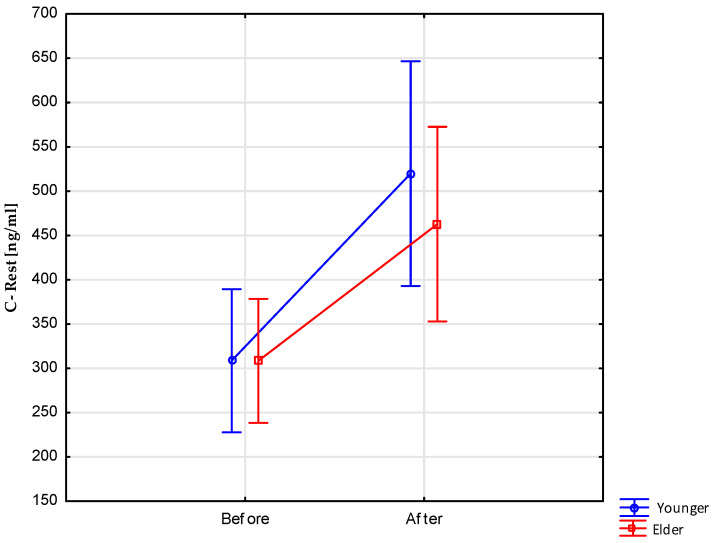
Resting cortisol levels before and after 8 days of water-only fasting.

Interaction effects for this measure were also not statistically significant, F(1.12) = 0.535, *p* > 0.05 (Figure 1).

Post hoc analyses additionally showed that statistically significant differences in the level of cortisol measured at rest occurred in subsequent measurements in both age groups (Table 3).

Subsequent measurements of the cortisol level before (319.89 ± 118.42 ng/mL) and after fasting (503.66 ± 159.36 ng/mL) measured after exercise also showed statistically significant differences, F(1.12) = 14,670, *p* < 0.05, η^2^ = 0.551. Post hoc analyses showed that the sources of these differences should be sought in the group of elder respondents (Table 3). At the same time, no significant differences were noted for the age groups: younger (417.82 ± 124.80 ng/mL) vs. elder (407.23 ± 145.96 ng/mL), F(1.12) = 0.028, *p* > 0.05. Interaction effects for this measurement were also insignificant, F(1.12) = 0.145, *p* > 0.05 (Table 3).

Studies have shown that fasting leads to a statistically significant increase in β-hydroxybutyrate concentration both at rest and during physical exertion (Table 3).

### 3.2. Psychological Measures

The subjective assessment of experienced stress, measured with the PSS 10 questionnaire by Cohen, Kamarck and Mermelstein, did not differentiate between age groups, F(1.12) = 0.437, *p* > 0.05, or values obtained before and after 8 days of water-only fasting, F(1.12) = 1.251, *p* > 0.05. Interaction effects, F(1.12) = 0.313, *p* > 0.05, also were not statistically significant, and the results for individual study groups were within the limits of reduced (4 stens) and low (3 stens) scores compared to the Polish population (Table 4).

Analyses referring to currently experienced anxiety (anxiety state) and anxiety in general (anxiety trait) before and after fasting did not show statistically significant differences for both anxiety state, F(1.12) = 0.037, *p* > 0.05, and for anxiety trait, F(1.12) = 2.576, *p* > 0.05. For both anxiety indices, no differences were detected in the examined age groups: state anxiety, F(1.12) = 0.419, *p* > 0.05, and trait anxiety, F(1.12) = 0.022, *p* > 0.05. The average results for the study groups, together with the corresponding sten scores, are presented in Table 5. As can be seen, the indications regarding the various forms of anxiety experienced by the subjects were within the low-score range in relation to the norms for the Polish population in the relevant age groups.

**Figure 2:**
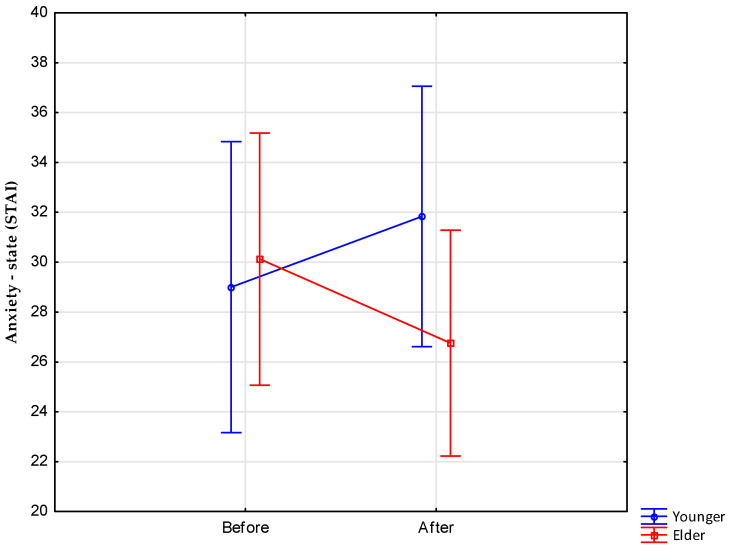
STAI results—interaction effects for the anxiety state variable and age grouping factors (younger vs. elder) and measurement (before vs. after fasting).

Further analyses of anxiety indices showed no significant interaction effects for the anxiety trait variable, F(1.12) = 0.022, *p* > 0.05, and significant interaction effects for the anxiety state variable, F(1.12) = 4.884, *p* < 0.05 (Figure 2).

This means that the level of anxiety state before fasting was lower in group younger than in group elder, and, after fasting, the intensity of anxiety state was the opposite—it was higher in group younger compared to group elder.

The adopted hypotheses assumed that fasting would affect not only the subjective experience of stress and anxiety, but also the efficiency of cognitive processes. To verify these hypotheses, the Color Connection Test (CTT) and the Culturally Neutral Cattell Intelligence Test CFT-20R were used. Analysis of variance for repeated measures and the grouping factor—age (younger vs. elder)—showed a statistically significant variability in the time needed to complete the task in the test (CTT-1) in subsequent measurements, F(1.12) = 5.403, *p* < 0.05, η^2^ = 0.310. The time taken by the subjects to complete this task was significantly shorter after fasting (37.51 ± 17.05 s) than before fasting (42.09 ± 13.53 s). At the same time, the time needed to complete the tasks by subjects from groups younger (34.28 ± 6.46 s) and elder (43.95 ± 18.91 s) did not differ significantly from a statistical standpoint, F(1.12) = 1.502, *p* > 0.05. There were also no significant interaction effects, F(1.12) = 1.216, *p* > 0.05. In both trials, the subjects basically did not make mistakes—there was only one error.

In the second part of the measurement, performed with the CTT-2 test, there were no significant differences in the task performance time before (80.30 ± 25.28 s) and after (75.11 ± 15.13 s) fasting, F(1.12) = 1.149, *p* > 0.05. However, differences were observed at the level of the statistical trend in the time taken to perform the task by younger subjects (68.03 ± 15.69 s) and older subjects (84.97 ± 20.54 s), F(1.12) = 3.219, *p* = 0.09. As in CTT-1, no significant interaction effects were observed in CTT-2, F(1.12) = 0.009, *p* > 0.05.

As for the other indicators of the performance of this task, no significant differences were observed in subsequent measurements. The number of numerical errors made was 2 for the first and second measurement. The results of color errors, which were identical in both measurements and amounted to 2, were similar.

Another analyzed indicator of the level of task performance in the CTT test was the so-called Interference Index1, which did not differentiate the study group either in the two measurements, F(1.12) = 0.915, *p* > 0.05, before (0.46 ± 0.10) and after fasting (0.50 ± 0.16), or by age, F(1.12) = 0.029, *p* > 0.05—Y (0.48 ± 0.14) vs. E (0.47 ± 0.12). Also in this case, similarly to the analyses for CTT-1 and CTT-2, there was no significant interaction effect, F(1.12) = 0.647, *p* > 0.05.

The results of the Culturally Neutral Cattell Intelligence Test version 2, revised by R. Wiess and B. Weiss (CFT-20R), did not show statistically significant differences between the measurement before (69.86 ± 9.99) and after (72.36 ± 11.76) fasting, F(1.12) = 2.588, *p* > 0.05, or between the younger (72.58 ± 9.47) and elder (70.00 ± 9.38) groups of the surveyed men, F(1.12) = 0.196, *p* > 0.05. There were also no significant interaction effects, F(1.12) = 0.750, *p* > 0.05.

In addition, strong correlations were found between the level of cortisol measured at rest before fasting and subjectively experienced stress and anxiety understood as a relatively constant feature. The higher the cortisol level, the lower the level of declared stress before fasting, the lower the intensity of the anxiety trait before and after fasting and the lower the intensity of the anxiety state after fasting. In addition, the declared level of stress before fasting correlated negatively with the level of resting and exercise stress after fasting.

In addition, the relationships between the cognitive performance indicators and the cortisol level only showed strong negative correlations between the time to complete the first task in the Connectivity Color Test (CTT-1). Other measures, such as fluid intelligence and time taken to perform tasks involving not only the ability to maintain attention, visual search and psychomotor skills, but also the ability to sequentially process information (CTT-2), did not reveal any significant associations with cortisol levels (Table 6).

For a more complete picture of the relationship between the subjective assessments of mental state and metabolic and hormonal changes observed before and after fasting, it should be added that no statistically significant correlations were obtained between the results of psychological tests PSS and STAI and the level of glucose and β-hydroxybutyrate.

The assessment of the intensity of stress before and after fasting negatively correlated with fluid intelligence; therefore, the greater the stress declared by the subject (PSS), the lower the scores obtained in the CFT-20R test. The state of stress, anxiety and fear significantly correlated with the time taken to perform simple tasks requiring concentration and graphomotor skills (CTT-1). Therefore, the higher the level of subjectively experienced stress and anxiety, the more time the subjects needed to complete this task (Table 7).

## 4. Discussion

The obtained subjective assessments of the mental state of the surveyed men should be considered in conjunction with the somatic and physiological changes occurring after 8 days of water-only fasting.

Undoubtedly, as expected, fasting had a positive effect on the physiological parameters of the subjects, such as blood pressure and heart rate, although it is worth noting that these variables were still within normal limits before the start of fasting. These values are significantly modified due to long-term fasting, which was confirmed in our studies, and, in the opinion of other authors, may also be caused by a change in the tension of the vegetative nervous system [49,50,51] and the development of adaptive mental reactions [52,53]. In addition, a significant 5.56 kg reduction in body mass on average was obtained, which was a beneficial effect of the applied fasting, as the subjects reached the upper limit of the physiological reference value for this variable, expressed by the BMI index, before the start of the experiment. In addition, in the final phase of fasting, a decrease in glucose levels was observed, with a simultaneous increase in the concentration of β-hydroxybutyrate, as an indicator of increasing ketosis [54]. There was also an increase in the level of cortisol measured at rest in both study groups (younger and elder) and an increase in the concentration of cortisol induced by exercise in the older group of men. These physiological changes gave grounds to expect adequate modifications in terms of subjective indications of stress, anxiety and fear, expressed in the results of our psychological tests, because glucose is the main substrate that nourishes the brain under physiological conditions [55]. β-hydroxybutyrate, together with another ketone body—acetoacetate, significantly nourishes the brain in a state of fasting [53,56,57], and the first of these compounds is an active signaling molecule for many cells [9,10], and cortisol is an important determinant of the body’s stress response [58]. In addition, animal studies have shown that fasting changes the composition of the intestinal microflora [59,60]. Liu et al. [61] proved that intermittent fasting enriches the composition of the gut microbiome and alters microbial metabolism, which improves cognitive functions related to spatial memory.

Meanwhile, our research did not record any significant effects indicative of psychological consequences of fasting, manifested by the level of perceived stress and anxiety in both groups, younger and elder. Interestingly, all indications of mental well-being were within the limits of reduced or low scores, which indicates the very good mental condition of the subjects before the start of fasting. The only statistically significant effect obtained in the study was the interaction effect (fasting–age) for the measurement of state anxiety in the STAI test. The level of state anxiety before fasting was lower in group younger than in group elder, and, after fasting, the level of state anxiety was the opposite—it was higher in the group of younger subjects compared to the older group.

These results may be an important clue for further research, in which the mental condition of the subjects before fasting should be controlled, and the age groups should be precisely selected. It can be assumed that the level and direction of changes in mental well-being depend on their initial qualification—a certain level of stress, fear and anxiety may be exacerbated by fasting, exceeding the body’s adaptive capacity. The intensification of negative affective states may be the result not so much of the level of stress experienced as the lack of adequate reserves of the body to cope with it. The interaction effect observed in the study could indicate the functioning of just such a mechanism among younger respondents.

The importance of the body’s resources to cope with stress caused by prolonged fasting for subjective mental well-being may also be demonstrated by the negative relationship between cortisol level and psychological indicators of stress and anxiety. Such negative correlations have been recorded between the level of cortisol and such pathological conditions as: generalized anxiety disorder, panic disorder or post-traumatic stress disorder [21]. Since the group of volunteers participating in our research did not have any somatic or mental diseases, exactly the opposite relationship was expected, i.e., a positive correlation between the level of anxiety and depression and serum cortisol concentration [20,62]. Considering the close relationships between the functional indicators of the nervous system and the metabolic and hormonal variables of the body, one should also expect significant correlations between the level of glucose or β-hydroxybutyrate and subjective measures of stress and anxiety. However, such relationships were not found in our research.

At the present stage of knowledge, it is impossible to unambiguously explain the negative correlations obtained in our research, or their absence, between the analyzed physiological and psychological indicators. It cannot be ruled out that the released cortisol activated the mechanisms of coping with stress and did not have a negative impact on the emotional and cognitive sphere. On the contrary, it had an activating and motivating effect in the discussed scope [31]. Perhaps the explanation for this effect lies with the subjects themselves and the group selection used. The low representativeness of the group of men in the studies presented by us may be evidenced by the low scores (expressed in stens) in tests measuring subjectively experienced stress and anxiety. It can also be assumed that the increase in negative emotions and the increase in stress levels occurred during the fasting, while the fact of its completion became a reason for the study participants to experience positive emotions [31].

Further considerations also cannot ignore the importance of motivation for positive self-presentation, expressed by the need for social approval, so the declared levels of stress and anxiety could have been falsified in the direction of better adaptation. It would be good to control this variable in further research by including a lie scale in the study.

Finally, it cannot be ruled out that, in the indications of subjective measures of mental well-being, the mechanism of post-decision dissonance reduction, described by social psychology, was important, the essence of which is to look for positive, not negative, consequences of one’s own choices—in this case, participation in the 8 days of water-only fasting [63]. Of course, this hypothesis should be verified in further research.

In terms of the cognitive abilities included in the study, improvement after fasting was noted only during the performance of simple tasks requiring concentration and graphomotor efficiency. Interestingly, the level of cortisol before the fasting negatively correlated with the time taken to perform motor tasks, while the level of cortisol after the fasting showed no relationship with the time taken to perform the task. Similarly, more cognitively advanced tasks and tasks requiring the participation of fluid intelligence, i.e., biologically conditioned (innate) intelligence, remained constant and independent of the impact of food shortages.

## 5. Conclusions

The presented data are the result of the first stage of a research project on the psychological consequences of long-term fasting, which caused significant somatic, metabolic and hormonal changes. On the other hand, mental well-being did not change under the influence of 8 days of the water-only fasting, and its relatively high level in these conditions was independent of physiological changes. The authors are aware of the numerous limitations of this study, but will use the results obtained in it for more comprehensive planning of subsequent experiments. Certainly, a major drawback of the described study was the relatively small size of the group. We realize that obtaining more volunteers will not be an easy task. Taking into account reports from similar studies, it is impossible not to notice that other authors struggled with similar problems [33,34]. It should also be mentioned that we deliberately did not group the men surveyed during the 8 days of water-only fasting in one place, isolated from the regular environment. We believe that these would have been artificial conditions, not corresponding to the preliminary study carried out before the start of the fasting. In our opinion, this would have been a methodological error, which would not have allowed the study to show the effects of hunger deprivation on the body. In addition, such a methodological procedure was also dictated by the fact that the volunteers participating in the research had previously participated in the fasting procedure many times and, at the same time, fulfilled their daily professional and family duties. It also seems advisable to use psychological methods in the subsequent stages that measure the currently experienced stress and mood but also the sense of achievement and effective self-control. We are convinced that special attention should be focused on explaining the relationship between physiological indicators of stress and its subjective perception, which was inconsistent with previous scientific reports. It seems that the mechanisms of effective coping with stress and regulating emotions may be of key importance to explain these relationships.

## Figures and Tables

**Table 1:** Somatic variables (M ± SD) before and after the 8 days of water-only fasting.

Variables	Age	Fasting		
		Before	After	*p*
Age (years)	Younger	38.50 ± 9.09	38.50 ± 9.09	>0.05
	Elder	54.00 ± 5.55	54.00 ± 5.55	>0.05
	Together	49.72 ± 13.03	49.72 ± 13.03	>0.05
BM (kg)	Younger	73.30 ± 11.15	67.83 ± 10.90	<0.001
	Elder	84.60 ± 8.21	78.96 ± 8.42	<0.001
	Together	79.75 ± 10.85	74.19 ± 10.79	<0.001
BH (cm)	Younger	178.17 ± 5.15	178.17 ± 5.15	>0.05
	Elder	178.87 ± 4.52	178.87 ± 4.52	>0.05
	Together	178.57 ± 4.62	178.57 ± 4.62	>0.05
BF (kg)	Younger	12.02 ± 5.61	9.90 ± 5.49	<0.001
	Elder	18.20 ± 4.82	16.41 ± 3.98	<0.001
	Together	15.55 ± 4.82	13.62 ± 5.60	<0.001
FFM (kg)	Younger	61.28 ± 6.30	57.63 ± 6.62	<0.001
	Elder	66.40 ± 4.28	62.55 ± 4.85	<0.001
	Together	64.21 ± 5.66	60.44 ± 5.99	<0.001
TBW (kg)	Younger	44.88 ± 4.62	42.42 ± 4.55	<0.001
	Elder	48.61 ± 3.12	45.79 ± 3.57	<0.001
	Together	47.01 ± 4.14	44.34 ± 4.22	<0.001
BMI (kg/m^2^)	Younger	23.11 ± 3.62	21.39 ± 3.51	<0.001
	Elder	26.44 ± 2.46	24.67 ± 2.45	<0.001
	Together	25.01 ± 3.35	23.26 ± 3.29	<0.001

**Table 2:** Variables of the circulatory system (M ± SD) along with the significance of differences in the studied age groups before and after the 8 days of water-only fasting.

Variables	Age	Fasting			Age		Fasting		Age vs. Fasting	
		Before	After	Together; Before–After	F	*p*	F	*p*	F	*p*
DBP-Rest (mmHg)	Younger	87.50 ± 8.80	80.00 ± 6.32	83.75 ± 7.56	2.243	>0.05	6.751	<0.05	0.468	>0.05
	Elder	79.38 ± 10.50	75.00 ± 9.64	77.19 ± 10.07						
	Together	82.86 ± 9.65	77.14 ± 7.96	^before–after = :0.027^						
DBP-Exercise (mm/Hg)	Younger	76.67 ± 10.80	75.83 ± 10.21	76.25 ± 10.50	0.0621	>0.05	0.0120	>0.05	0,3190	>0.05
	Elder	74.36 ± 10.16	75.36 ± 8.63	75.00 ± 9.39						
	Together	75.36 ± 10.32	75.71 ± 9.64							
SBP-Rest (mmHg)	Younger	130.00 ± 8.37	118.33 ± 10.33	124.17 ± 9.35	3.474	>0.05	22.374	<0.001	0.077	>0.05
	Elder	120.62 ± 14.25	107.50 ± 15.34 ^b^	114.06 ± 14.79						
	Together	124.64 ± 12.63	112.14 ± 11.39 ^b = 0.0192^	^before–after = 0.0004^						
SBP-Exercise (mmHg0)	Younger	192.50 ± 28.42	166.67 ± 22.73	179.58 ± 25.57	0.0503	>0.05	6.179	<0.05	0.847	>0.05
	Elder	180.00 ± 14.39	168.12 ± 15.34	174.06 ± 14.86						
	Together	185.36 ± 21.52	167.50 ± 18.05	^before–after = :0.035^						
HR-Rest (bpm)	Younger	63.17 ± 8.40	71.17 ± 14.63	67.17 ± 11.51	0.0669	>0.05	5.1732	<0.05	0.1530	>0.05
	Elder	65.75 ± 9.74	71.50 ± 13.15	68.83 ± 11.44						
	Together	64.64 ± 8.94	71.34 ± 13.25	^before^ ^–after = 0.036^						
HR-Exercise (bpm)	Younger	158.00 ± 23.55	158.17 ± 19.67	158.33 ± 21.61	0.758	>0.05	0.168	>0.05	0.375	>0.05
	Elder	167.13 ± 12.02	163.75 ± 9.66	165.44 ± 10.84						
	Together	163.21 ± 17.69	161.57 ± 14.35							

M—arithmetic means; DBP—diastolic blood pressure; SBP—systolic blood pressure; HR—heart rate; ^b^—before 8 days of water-only fasting vs. after 8 days of water-only fasting (group younger).

**Table 3:** Metabolic and hormonal variables (M ± SD) along with the significance of differences in the studied age groups before and after 8 days of water-only fasting.

Variables	Age	Fasting			Age		Fasting		Age vs. Fasting	
		Before	After	Together Before–After	F	*p*	F	*p*	F	*p*
Glucose-Rest (mmol/L)	Younger	4.68 ± 0.36	3.76 ± 0.30 ^a^	4.22 ± 0.33	1.489	>0.05	35.651	<0.001	0.344	>0.05
	Elder	5.11 ± 0.58	3.99 ± 0.85 ^b^	4.55 ± 0.71						
	Together	4.93 ± 0.53 ^a = 0.0295^	3.90 ± 0.66 ^b = 0.000052^	^before–after = 0.000052^						
Glucose-Exercise (mmol/L)	Younger	5.64 ± 0.89	4.28 ± 0.45 ^a^	4.96 ± 0.67	1.549	>0.05	25.823	<0.001	0.055	>0.05
	Elder	5.98 ± 0.72 ^a = 0.0317^	4.74 ± 0.86 ^b^	5.36 ± 0.79						
	Together	5.84 ± 0.78 ^a = 0.0317^	4.54 ± 0.73 ^b = 0.02348^	^before–after = 0.00026^						
Cortisol-Rest (ng/mL)	Younger	308.68 ± 89.24	519.85 ± 164.15 ^b^	414.27 ± 99.21	0.306	>0.05	22.193	<0.001	0.535	>0.05
	Elder	308.50 ± 93.24	462.89 ± 105.18 ^a^	385.6 ± 126.709						
	Together	308.58 ± 87.36 ^a = 0.06^	487.30 ± 140.07 ^b = 0.03^							
Cortisol- Exercise (ng/mL)	Younger	336.18 ± 89.32	499.45 ± 202.60	417.82 ± 124.80	0.028	>0.05	14.670	<0.05	0.145	>0.05
	Elder	307.63 ± 157.27	506.83 ± 92.34 ^a^	407.23 ± 145.96						
	Together	319.89 ± 118.42 ^a = 0.05^	503.66 ± 159.36							
β-hydroxybutyrate-Rest (mmol/L)	Younger	0.318 ± 0.22	4.40 ± 0.55	2.36 ± 0.38	0.096	>0.05	86.357	<0.001	0.1008	>0.05
	Elder	0.315 ± 0.20	^4.13 ± 1.98^	2.22 ± 1.1						
	Together	0.317 ± 0.20	4.25 ± 1.50	2.28 ± 0.85						
β-hydroxybutyrate-Exercise (mmol/L)	Younger	0.24 ± 0.15	3.96 ± 0.95	2.1 ± 0.55	0.682	>0.05	82.697	<0.001	0.57	>0.05
	Elder	0.20 ± 0.17	3.33 ± 1.68	1.76 ± 0.92						
	Together	0.21 ± 0.16	3.60 ± 1.40	1.90 ± 0.78						

M—arithmetic means; ^a^—before 8 days of water-only fasting vs. after 8 days of water-only fasting (group elder); ^b^—before 8 days of water-only fasting vs. after 8 days of water-only fasting (group younger).

**Table 4:** Results (M ± SD) of the PSS 10 (Perceived Stress Scale) test: comparison of groups younger and elder.

	Younger	Stens	Elder	Stens	*p*
Before fasting	10.50 ± 4.23	4	9.50 ± 5.73	4	>0.05
After fasting	10.00 ± 3.41	4	8.00 ± 4.00	3	>0.05
*p*	>0.05		>0.05		

M—arithmetic means. Note the use of the conversion scale (stens) makes it possible to determine the level of results obtained for individual questionnaires in relation to the population constituting the standardization group. The sten scale ranges from 1 to 10, with 1 being very low, 10 being very high, and 5–6 being normal.

**Table 5:** STAI (State-Trait Anxiety Inventory) score (M ± SD): comparison of groups younger and elder.

	Anxiety State					Anxiety Trait				
	Younger	Stens	Elder	Stens	*p*	Younger	Stens	Elder	Stens	*p*
Before fasting	29.00 ± 4.90	3	30.12 ± 7.53	4	>0.05	35.00 ± 9.53	4	34.00 ± 3.66	3	>0.05
After fasting	31.82 ± 5.49	4	26.75 ± 6.14	3	>0.05	32.50 ± 6.35	3	32.50 ± 6.78	2	>0.05
*p*	>0.05		>0.05			>0.05		>0.05		

**Table 6:** Correlation coefficients between the indicators of perceived stress and anxiety, as well as cognitive performance and the level of cortisol.

Stress and Anxiety Indicators	Cortisol—Before Fasting		Cortisol—After Fasting	
Before Fasting	Rest	Exercise	Rest	Exercise
PSS	**−0.54**	−0.15	**−0.59**	**−0.54**
STAI–State	−0.44	−0.10	−0.31	−0.29
STAI–Trait	**−0.67**	−0.28	−0.49	−0.25
After fasting				
PSS	−0.41	−0.03	−0.43	−0.16
STAI–State	**−0.57**	−0.24	−0.15	−0.25
STAI–Trait	**−0.69**	−0.25	−0.32	−0.13
Efficiency indicators of cognitive processes	C—Before fasting		C—After fasting	
Before fasting	Rest	Exercise	Rest	Exercise
CFT-20R	0.25	0.01	0.06	−0.13
CTT-1 time	**−0.62**	−0.20	−0.49	−0.29
CTT-2 time	−0.45	−0.18	−0.49	−0.37
CTT-Interference index	0.20	0.04	−0.02	−0.10
After fasting				
CFT-20R	0.34	−0.05	0.25	0.14
CTT-1 time	**−0.54**	−0.15	−0.39	−0.14
CTT-2 time	−0.36	−0.12	−0.36	−0.16
CTT-Interference index	0.50	0.17	0.25	0.10

Note: correlations with *p* < 0.05 are bolded.

**Table 7:** Correlation coefficients between subjective indicators of stress and anxiety and cognitive performance measured before and after fasting.

Indicators of Efficiency of Cognitive Processes	Stress and Anxiety Indicators Before Fasting			Stress and Anxiety Indicators After Fasting		
Before Fasting:	PSS	STAI-State	STAI-Trait	PSS	STAI-State	STAI-Trait
CFT-20R	**−0.55**	−0.48	−0.03	**−0.60**	−0.21	−0.30
CTT-1 time	**0.55**	**0.53**	0.35	**0.54**	0.44	**0.65**
CTT-2 time	0.48	0.35	0.13	0.37	0.20	0.32
CTT-Interference index	−0.14	−0.30	−0.30	−0.33	−0.41	−0.50
After Fasting:						
CFT-20R	**−0.61**	−0.48	−0.09	−0.51	−0.12	−0.32
CTT-1 time	0.32	0.42	0.43	0.42	0.45	**0.71**
CTT-2 time	0.38	0.29	0.08	0.31	0.05	0.43
CTT-Interference index	−0.19	−0.31	**−0.55**	−0.30	**−0.56**	**−0.63**

Note: correlations with *p* < 0.05 are bolded.

## Data Availability

All the data are available from the first author under reasonable request.
